# Survival Rate of Breast Cancer Based on Geographical Variation in Iran, a National Study

**DOI:** 10.5812/ircmj.3631

**Published:** 2012-12-06

**Authors:** Mohammad Movahedi, Shahpar Haghighat, Maryam Khayamzadeh, Afshin Moradi, Ali Ghanbari-Motlagh, Hamidreza Mirzaei, Mohammad Esmail-Akbari

**Affiliations:** 1 Cancer Research Centre, Shahid Beheshti University of Medical Science, Tehran, IR Iran; 2 Epidemiology Department, School of Public Health, ShahidBeheshti University of Medical Science, Tehran, IR Iran; 3 Quality of Life in Cancer Department, Iranian Center for Breast Cancer, ACECR, Tehran, IR Iran

**Keywords:** Breast Neoplasm, Survival Rate, Pathologic Type, Place of Residence, Iran

## Abstract

**Background:**

Breast cancer is the most common cancer among women worldwide. Based on the latest Iranian national cancer department report, the total number of women registered with breast cancer was 6976 cases during 2007. Five year survival is one of the indicators used for evaluation of the quality for care to different types of malignancies including breast cancer.

**Objectives:**

The aim of this study was to estimate survival rate of breast cancer in 6147 Iranian patients at a national level in different geographic regions.

**Materials and Methods:**

6147 cases of breast cancer, which had telephone number and were diagnosed between 2001-2006, were called to obtain information about their life status. Survival estimates were calculated using the Kaplan-Meier method, and the survival probability was calculated for the overall cohort and in different categories of gender, age and pathologic type of tumor. Hazard ratios (HR) according to demographic and risk variables were calculated by Cox's proportional hazard model.

**Results:**

The overall 5-year survival rate was 71.0%. The mean survival time was different between men and women, which was statistically significant. The number of men involved with breast cancer was 172 (2.8%) of all cases. The 5-year survival rate for patients in age group 41-50 years was significantly higher than other age groups (P = 0.001). The likelihood of death was higher in patients with 61 years old or more years rather than those below forty years old (HR = 1.31; 95% CI: 1.12-1.55).

**Conclusions:**

The findings of this study might help Iranian health managers: 1) to be more conscious about geographical and regional determinants which will affect overall survival rate. 2) To carry preventive activities such as public education particularly in Iranian men. 3) To think about screening and early detection of breast cancer.

## 1. Background

Breast cancer is the most common cancer among women across the globe. The estimated number for incident cases in developed and developing countries were 636,000 and 514,000 respectively during 2002. This cancer is also the most important cause of neoplastic deaths among women with 458,000 deaths in 2008 worldwide. In terms of time trends for breast cancer incidence and mortality, the incidence has increased rapidly during the last decades in many developing countries compared with slow increase of this cancer in developed countries (1).

Breast cancer is the most incident cancer and the fifth cause of death due to malignancies among Iranian women (2) with approximately 8500 incident cases per year. The burden of breast cancer is also high with 17,772 years life lost due to this disease. The breast cancer rate is 11 percent of the whole malignancies among both genders (3). Based on the latest MOH & ME cancer registration report, the total number of women with a new breast cancer was 6,976 (Age Standardized Rate = 27.15 per 105) during 2007 (with expected number of 8500 patients), 78 % of which was infiltrating ductal carcinoma (4). A systematic review carried out by Akbari found the incidence of breast cancer in women was 30 per 100,000 person- years in Isfahan province in the center of Iran (5).

This cancer affects Iranian women at least one decade younger than their counterparts in developed countries (6).The mortality rate of breast cancer was 5.8 per 100,000 women in Tehran in 1998 (7), 2.5 per 100,000 for female population. Estimation of five-year survival is one of the indicators used to evaluate the quality of care for different types of malignancies. Iran has a total population of just over 70 million and to our knowledge some studies have been done already to estimate survival rate of breast cancer in different regions of Iran with limited cases, which could not be representative to breast cancer survival rate at a national level. A study conducted in Southern Iran, investigated the association between the survival and socio-demographic and pathologic factors in 1,148 women, found a five-year overall survival rate of 58% (95%CI; 53%–62%) (8). They also showed that high family income, smoking, tumor size and grade, and number of involved nodes were significantly related to survival rate.

In a screening project of 52,200 women in Shahre-Kord, a province in the center of Iran, 40 breast cancer cases were detected, 31 cases (77.5%) of whom survived during 6 years of follow-up (6).The overall relative five-year survival rate was found to be 62% (SE = 0.04) in a study conducted on 167 breast cancer patients in 1997 (7). Receiving neo-adjuvant chemotherapy (as the initial treatment) was an independent predictor of poorer survival (Hazard ratio = 4.56, 95% CI 2.20-9.44, P < 0.0001). The other variables (older age and late stage disease), although associated with high hazard rates, were not significantly related to survival.

Akbari, et al followed up 441 breast cancer patients between 1994- 2007. Five and ten-year survival for all cases was 81 % and 77% respectively. They concluded that overall five-year survival was comparable with developed countries with different health delivery system and different ages and stages of the disease (9).Other studies have also tried to estimate the five-year survival rate of breast cancer in various cities in Iran; 70% in Shiraz (10), 89% in Baghiyatollah hospital of Tehran (11), 58% in Semnan (12).

Sadjadi, et al. compared breast cancer survival between two populations: Ardabil (North west of Iran), Iran and British Columbia, Canada. The age-standardized one-year relative survival rates in BC were 0.99 (SE = 0.004) for adult women younger than age 50 and 0.97 (SE = 0.012) for women aged 50 or more. The age-standardized one-year relative survival rate in Ardabil was 0.92 (SE = 0.020) for adult women younger than age 50 and 0.95 (SE = 0.037) for women aged 50 or more. They have concluded that breast cancer patients in British Columbia had a better one-year survival rates than patients in Ardabil overall and for each age group less than 60 (13). Due to the importance of managing breast cancer at a national level, it is important to understand the epidemiology of this disease including incidence, prevalence and survival rate more precisely.

## 2. Objectives

This study was conducted to clarify the survival rate of breast cancer patients among Iranian cases who were diagnosed between 2001 and 2006 and followed until 2009.

## 3. Materials and Methods

The pathology based cancer reports published by National Cancer Department in Ministry of Health and Medical Education (MOH&ME) was used as the main source of incident data for breast cancer in Iran from 2001 to 2006. The coverage rate of cancer registration in 2006 was 83%, while this value was less than 40% in the first year (2001) of cancer registration establishment (2, 4,14-17).

### 3.1. Statistical Analysis

Overall, 25,618 cases were identified as breast cancer between years 2001 and 2006 of which the telephone numbers were available for 12,321 (44.6%).Due to some difficulties in telephone registries and changing addresses, we accessed 6,147 cases (24% of all cases). A trained nurse called this study group and interviewed with them or their family and collected data about health status of patients. To find whether the study cases are representative of target population, their age group and gender and also the pathologic type distribution were compared with those of the whole 25618 cases across the country as the target population.

For statistical analyses, the continuous quantitative variable of age was categorized into four age groups: less than 40 year, 41-50, 51-60 and 61 or more. According to the number of patients in each pathologic type, the patients were categorized into 6 main groups: Carcinoma Insitu, Paget, Infiltrating Ductul Carcinoma (IDC), Infiltrating Lobular Carcinoma (ILC), Mixed (combination of two IDC and ILC) and "others". The latter category consisted of Carcinoma, Sarcoma, and Lymphoma. Patients’ residential locations were categorized into nine geographical regions based on similar socioeconomic status that are recommended by MOH and ME. Number of days between diagnosis and date of death or end time of the study (Dec 2009) was considered as follow-up time. By definition, survival means the time extend from diagnosis to the time of death or last visit.

Survival rates were calculated using the Kaplan-Meier method, and the survival probability was estimated for overall cohorts and also for different categories of age, gender and pathologic type. The significant differences in survival rates among gender and age group classifications were tested by the log rank test for trends. Hazard ratios (HRs) according to demographic and risk variables were calculated by Cox's proportional hazard model, with 95% Confidence Interval (CI). Four variables of gender, pathologic type and age groups and place of residence were entered in Cox's model for multivariate analysis. The log minus log plotted against survival time for each covariate did not show any deviation from the proportionality assumption. The data were analyzed with SPSS V.17.0. Significance levels were considered by two tailed tests with P < 0.05.

## 4. Results

Analysis was done on 6,147 breast cancer patients diagnosed between 2001 and 2006. About 80.4% of cases (4,940 patients) were alive during the follow-up time. The longest follow-up time was 2,646 days. Table 1 compares the baseline characteristics of the study subjects (n = 6147) with target population (n = 25618). 5,975 (97.2%)cases were women and 172 (2.8%) cases were men. The mean age was 49.84 ± 12.36 years. Half of patients were 48 years old and younger. The most common age group of patients was 41- 49 years. The most common pathologic type of tumor in both men and women was IDC (Invasive Ductal carcinoma) (Table 1).

**Table 1:** Comparing the Basic Characteristics of Target Population and Study Subjects of Breast Cancer

	Study subjects, No. (%)	Target population, No. (%)	*P* value
**Gender**			0.6
Female	5975(97.2%)	24833 (96.9%)	
Male	172 (2.80%)	785 (3.1%)	
**Age groups**			0.6
≤ 40 years	1417 (23.1%)	6287(24.5%)	
41-50 years	2119 (34.5%)	8687 (33.9%)	
51-60 years	1435 (23.3%)	5799 (22.7%)	
≥ 61 years	1143 (18.6%)	4845 (18.9%)	
Missing	33 (0.5%)	-	
**Pathologic type**			0.8
Insitu	195(3.20%)	805 (3.10%)	
Paget	62 (1.00%)	232 (0.90%)	
IDC	5499 (89.5%)	22957 (89.6%)	
ILC	331 (5.40%)	1303 (5.20%)	
Mixed	16 (0.30%)	61 (0.20%)	
**Others**	44 (0.72%)	260 (1.00%)	

**Figure 1:**
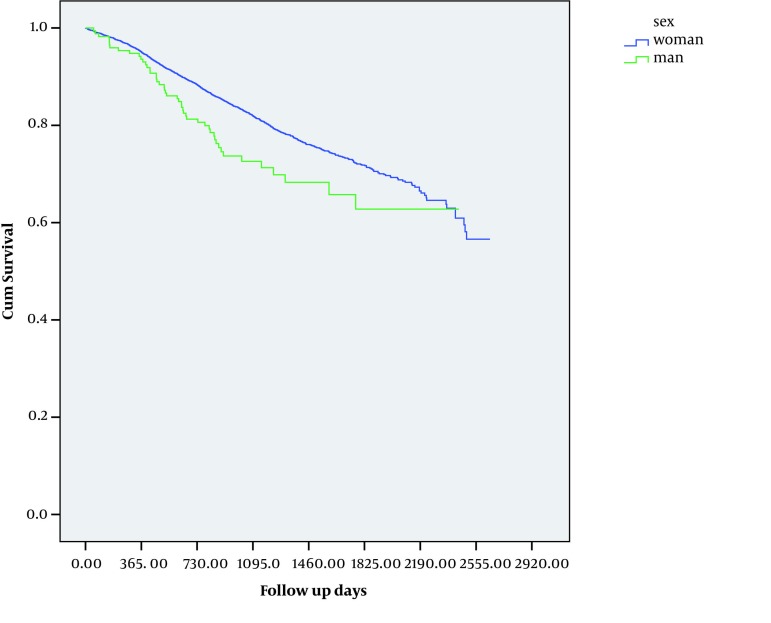
The Kaplan-Mayer Overall Survival Curve of Breast Cancer by Gender

Comparison between study subjects and target population of breast cancer by pathologic type and gender has been presented in Table 2. The distribution of the pathological features among men and women were different and Lymphoma was the most common type (42.8%) of "others" category in men.The crude 1 year, 2 years, 3 years, 4 years and 5 years survival rate for the whole cohorts was 95.0%, 88.0%, 82.0%, 75.0% and 71.0 % respectively. The mean survival time was 2091 and 1831 days for women and men respectively. This difference was statistically significant (P = 0.004). The five-year survival rate for women and men was 72.0% and 60.0% respectively (Figure 1). The five-year survival rate for patients of 41-50 years was significantly higher than other age groups (P < 0.001). Different pathologic types of tumors showed no significant correlation with survival rate of patients. Table 3 shows the survival rate of breast cancer by residential region. The lowest five-year survival rate was found among patients who lived in northwest region of the country (62.1%) and the highest one was among residents of the upper part of West South region (76.2%). This inter-regional variation was statistically significant ( P < 0.05). The differences of mean age between these 9 regions were statistically significant (P < 0.001). According to Scheffe Post Hoc tests this significance was due to difference between patients' mean age of northwest region (47.5 ± 11.58) and central region (north part) (50.9 ± 12.28). The distribution of pathologic tumor types was different between these regions, too (P = 0.007). The highest frequency of in situ carcinoma (6.1%) had been recorded in Middle West region and the lowest one (2.3%) in central region (south part).The univariate and multivariate analyses for different variables are shown in Table 4.Patients in age group more than 60 years had a worse prognosis than those of forty years old and younger(HR = 1.31; 95% CI: 1.12-1.55).

**Table 2:** Distribution of Study subjects and Target Population of Breast Cancer Patients by Pathologic Type and Gender

	Study subjects		Target population	
	Female, No. (%)	Male, No. (%)	Female, No. (%)	Male, No. (%)
**In situ**	192 (3.20%)	3 (1.74%)	784 (3.2%)	21 (2.7 %)
**Paget**	60 (1.00%)	2 (1.16%)	229 (0.92%)	3 (0.38%)
**IDC**	5348 (89.5%)	151 (87.8%)	22244 (89.6%)	713 (90.8%)
**ILC**	323 (5.41%)	8 (4.70%)	1284 (5.1%)	19 (2.4%)
**Mixed**	16 (0.30%)	0	61 (0.25%)	0
**Others**	36 (0.60%)	8 (4.70%)	231 (0.93%)	29 (3.7%)
**Others Subtype**				
**Sarcoma**	19 (51.4%)	2 (28.6%)	124 (53.7%)	11 (37.9%)
**Lymphoma**	19 (27.0%)	3 (42.8%)	61 (26.4%)	11 (37.9%)
**Unknown**	8 (21.6%)	3 (29.6%)	46 (19.9%)	7 (24.1%)
**Total**	5975 (100%)	172 (100%)	24833 (100%)	785 (100%)

**Table 3:** Survival Rate of Breast Cancer by Place of Residence

Place of residence (Region)	No. (%)	One year Survival (%) ± SE	Two year Survival (%) ± SE	Three year Survival (%) ± SE	Four year Survival (%) ± SE	Five year Survival (%) ± SE
**West South (Upper part)**	728 (11.8%)	94.4 ± 0.009	87.5 ± 0.013	82.9 ± 0.016	78.3 ± 0.021	76.2 ± 0.025
**Central (North part)**	625 (10.2%)	95.2 ± 0.005	88.5 ± 0.007	82.8 ± 0.009	77.9 ± 0.012	75.3 ± 0.015
**Central (Center part)**	359 (5.8%)	94.8 ± 0.007	88.3 ± 0.01	82.7 ± 0.013	76.4 ± 0.016	73.3 ± 0.018
**Middle West**	437 (7.1%)	91.9 ± 0.014	86.5 ± 0.018	76.3 ± 0.026	73.6 ± 0.029	69.6 ± 0.036
**North**	294 (4.8%)	96.3 ± 0.007	88.1 ± 0.012	80.6 ± 0.017	75.7 ± 0.021	69.3 ± 0.028
**South West (Lower part)**	1974 (32.1%)	95.2 ± 0.01	88.2 ± 0.016	82.8 ± 0.02	76.1 ± 0.025	68.9 ± 0.032
**Central (South part)**	221 (3.6%)	96.4 ± 0.013	90.9 ± 0.016	81.9 ± 0.029	74.1 ± 0.040	67.4 ± 0.052
**East**	430 (7.0%)	95.3 ± 0.01	87.6 ± 0.016	80.4 ± 0.021	72 ± 0.032	65.7 ± 0.046
**North West**	1078 (17.6%)	92.5 ± 0.015	84.2 ± 0.02	76.0 ± 0.028	65.5 ± 0.038	62.1 ± 0.043

**Table 4:** Hazard Ratios (95% CI) Estimated From Univariate and Multivariate Survival Analysisof Breast Cancer

	HR (95% CI) Univariate	HR (95% CI) Multivariate
**Gender**		
Female	1.00	1.00
Male	1.51 (1.14-2.10)	1.33 (0.99-1.78)
**Age groups**		
40 years or less	1.00	1.00
41-50 years	0.68 (0.58-0.78)	0.68 (0.58-0.79)
51-60 years	0.94 (0.80-1.11)	0.95 (0.81-1.12)
61 years or more	1.31 (1.12-1.54)	1.31 (1.12-1.55)
**Pathologic type**		
In situ	1.00	1.00
Paget	1.10 (0.56-2.10)	1.14 (0.59-2.19)
IDC	1.17 (0.83-1.64)	1.17 (0.83-1.65)
ILC	1.12(0.73-1.70)	1.19 (0.78-1.81)
Mixed	1.00(0.24-4.18)	1.02 (0.24-4.24)
Others	0.94(0.43-2.02)	0.77(0.35-1.68)
**Place of residence**		
North	1.00	0.86(0.68-1.13)
West South	0.93(0.7-1.16)	1
Middle West	1.14(0.86-1.5)	1.13(0.86-1.49)
South West	1.04(0.81-1.35)	1.04(0.8-1.34)
North West	1.4(1.07-1.85)	1.43(0.1.08-1.88)
Central (North part)	0.91(0.75-1.11)	0.88(0.73-1.08)
Central (south part)	1.009(0.72-1.41)	0.97(0.69-1.37)
East	1.08(0.83-1.41)	1.06(0.81-1.39)
Central (Center part)	0.94(0.76-1.16)	0.92(0.74-1.14)

## 5. Discussion

The survival rate of breast cancer cases is an important issue to help managers improve the quality of care for patients. It can also guide physicians and health personnel to promote their approach in managing breast cancer cases considering the last outcome of the care. The findings of this study showed that overall five-year survival rate of breast cancer throughout the country was 71%. This figure varied (from 76.2% to 62.1%) based on different data obtained from different geographical regions. These varieties may be due to multiple factors such as disease staging, knowledge and attitude, geographical and ethnical issues, socioeconomic factors and finally quality of care by care givers. This finding is also in agreement with findings of a study conducted by Taioli 2010 which showed the relation between the place of birth and residence, and survival rate of breast cancer (18). Another study conducted by Sadjadi compared survival rate of breast cancer between two populations of Ardebil and British Columbia. Their findings showed that BC patients survived one year more than Ardebil cases (13).

Family income in upper part of West South (maximum survival rate) is the highest and the the North West (minimum survival rate) is ranked third. Moreover, North West with the lowest survival rate had the lowest mean age of patients and the highest prevalence of invasive ductal carcinoma. Comparing mean age of the patients, pathology type, literacy and employment, there were no statistically significant difference between areas with maximum and minimum survival rates. High survival rate of upper part of West South may be due to a screening program since 1994, which led to a downstage for breast cancer.

Breast cancer survival has slowly increased in developed countries, where it now reacheses 85%, following improvements in screening practices and treatments. On the other hand, survival rate in developing countries remains around 50-60% (1). As Akbari et al mentioned ,the five-year survival rate was 81% and ten-year was 77%. A study on private breast cancer institute data showed that five-year survival rate is 89 %, which is comparable with developed countries (9).From 25618 breast cancer patients, only 6147 cases were followed up throughout the country (24%). However, there was no statistically significant difference between the study subjects and target population in terms of gender, age group, and pathologic type distribution of cases. So the subjects might be considered as a representative sample of the whole target population of patients with breast cancer across the country (Table 1).

According to the results of this study, the mean age of breast cancer in Iran was 49 years and the most common pathologic type in both genders was IDC. This is in agreement with other studies, which have been done in different parts of country (3, 10, 11). Iranian Breast cancer cases are one decade younger than their western counterparts (6); Young cases will affect the diagnosis and treatment approach and ought to be considered by health managers. The higher percentage (3.1 %) of Iranian men affected by breast cancer compared to worldwide average is another important issue. This finding was confirmed by a descriptive study in Babolsar, Iran, where during 5 years, 403 breast cancer patients were studied and the prevalence of breast cancer in men was 1.7%, which was higher than other countries (19). Study findings also showed that lymphoma was the most common type (42.7%) of "others" category in men compared to 27 percent in women. These findings might suggest the necessity of further planning on male education as one of the primary prevention strategies.

Factors of age and gender were associated with survival rate of breast cancer after adjusting for other factors of this study. Increasing age in both genders was found to be a poor prognostic factor for survival rate of breast cancer. These findings replicate findings of other studies conducted in Iran (7, 10, 12). However, one study in southern part of Iran did not support the relationship between younger age and survival rate (8). Morover, another study introduced age as independent factor correlated with poor prognosis of breast cancer in women less than 35 years old (20); different categorization and setting for age might be an explanation for this difference. On the other hand, data was obtained from National Cancer Department in Ministry of Health, which is pathologic-based. The coverage rate of cancer in this department varies between 40 and 83 percent. Thus, there is some possibility of existence of selection bias for this study. In terms of histopathology types of breast cancer, since various pathologists have reported them across the country, inter-observation variation is another possible limitation for this study, which might affect the results.

Owing to limitation of data record, we were not able to evaluate the impact of other important factors such as staging, tumor size, grading on survival rate in this relatively large study, the subject that remains to be explored by future studies.Analysis showed variation of overall survivals in different regions of Iran (Table 3). It may be due to some socioeconomic factors (Table 5) and mean age of the patients. But alongside social determinants, the quality of care may be an important effective element for the difference of overall survival rates.

**Table 5:** Geographic Areas Classification and Socioeconomic Factors

	Literacy (%)	Employment (%)	Population urban/rural (ratio)	Family income per year (Rials)
**North**	84.69	88.25	1.18	400432825
**South West (upper)**	84.26	85.35	1.47	469933897
**Middle West**	82.36	86.8	1.59	344053670
**South West (lower)**	82.35	84.85	1.15	212647199
**North west**	80.27	88.05	1.65	404110465
**Center (north)**	86.75	87.22	7.4	409266719
**Center (south)**	75.41	86.07	1.18	156344174
**East**	82.14	90.6	1.80	139629736
**Center (Central)**	86.4	86.2	3.65	275838949

The findings of this study might help Iranian health managers and specialists: 1) to be more concerned about quality of care and different social determinants in different parts of the country. 2) To be more conscious about male breast cancer in number and pathologic features. 3) Early diagnosis may have important role in down-staging and increasing survival rate as we have shown in some part of country with implementation screening or educational program. 4) A variety of factors are likely to affect the survival rate such as sex, age, geographical residence, socioeconomic factors and quality of care.
